# ChatGPT Performance Deteriorated in Patients with Comorbidities When Providing Cardiological Therapeutic Consultations

**DOI:** 10.3390/healthcare13131598

**Published:** 2025-07-03

**Authors:** Wen-Rui Hao, Chun-Chao Chen, Kuan Chen, Long-Chen Li, Chun-Chih Chiu, Tsung-Yeh Yang, Hung-Chang Jong, Hsuan-Chia Yang, Chih-Wei Huang, Ju-Chi Liu, Yu-Chuan (Jack) Li

**Affiliations:** 1 Taipei Heart Institute, Taipei Medical University, Taipei 11002, Taiwan; b8501043@tmu.edu.tw (W.-R.H.); b101092035@tmu.edu.tw (C.-C.C.); 17257@s.tmu.edu.tw (C.-C.C.); 15535@s.tmu.edu.tw (T.-Y.Y.); 18403@s.tmu.edu.tw (H.-C.J.); 2 Division of Cardiology, Department of Internal Medicine, School of Medicine, College of Medicine, Taipei Medical University, Taipei 11002, Taiwan; 3 Division of Cardiology, Department of Internal Medicine, Shuang Ho Hospital, Taipei Medical University, New Taipei City 23561, Taiwan; 4 School of Medicine, College of Medicine, Taipei Medical University, Taipei 11002, Taiwan; jc985137@gmail.com; 5 Department of Biochemistry and Molecular Biology, Johns Hopkins Bloomberg School of Public Health, Baltimore, MD 21205, USA; lli149@jhu.edu; 6 Clinical Big Data Research Center, Taipei Medical University Hospital, Taipei Medical University, Taipei 110301, Taiwan; lovejog@tmu.edu.tw; 7 Graduate Institute of Biomedical Informatics, College of Medical Science and Technology, Taipei Medical University, Taipei 106339, Taiwan; gracehuang@tmu.edu.tw; 8 International Center for Health Information Technology (ICHIT), College of Medical Science and Technology, Taipei Medical University, Taipei 106339, Taiwan; 9 Research Center of Big Data and Meta-Analysis, Wan Fang Hospital, Taipei Medical University, Taipei 116079, Taiwan; 10 Department of Dermatology, Taipei Municipal Wanfang Hospital, Taipei 116, Taiwan

**Keywords:** ChatGPT, large language model, cardiovascular disease

## Abstract

**Background**: Large language models (LLMs) like ChatGPT are increasingly being explored for medical applications. However, their reliability in providing medication advice for patients with complex clinical situations, particularly those with multiple comorbidities, remains uncertain and under-investigated. This study aimed to systematically evaluate the performance, consistency, and safety of ChatGPT in generating medication recommendations for complex cardiovascular disease (CVD) scenarios. **Methods**: In this simulation-based study (21 January–1 February 2024), ChatGPT 3.5 and 4.0 were prompted 10 times for each of 25 scenarios, representing five common CVDs paired with five major comorbidities. A panel of five cardiologists independently classified each unique drug recommendation as “high priority” or “low priority”. Key metrics included physician approval rates, the proportion of high-priority recommendations, response consistency (Jaccard similarity index), and error pattern analysis. Statistical comparisons were made using Z-tests, chi-square tests, and Wilcoxon Signed-Rank tests. **Results**: The overall physician approval rate for GPT-4 (86.90%) was modestly but significantly higher than that for GPT-3.5 (85.06%; *p* = 0.0476) based on aggregated data. However, a more rigorous paired-scenario analysis of high-priority recommendations revealed no statistically significant difference between the models (*p* = 0.407), indicating the advantage is not systematic. A chi-square test confirmed significant differences in error patterns (*p* < 0.001); notably, GPT-4 more frequently recommended contraindicated drugs in high-risk scenarios. Inter-model consistency was low (mean Jaccard index = 0.42), showing the models often provide different advice. **Conclusions**: While demonstrating high overall physician approval rates, current LLMs exhibit inconsistent performance and pose significant safety risks when providing medication advice for complex CVD cases. Their reliability does not yet meet the standards for autonomous clinical application. Future work must focus on leveraging real-world data for validation and developing domain-specific, fine-tuned models to enhance safety and accuracy. Until then, vigilant professional oversight is indispensable.

## 1. Introduction

Cardiovascular diseases (CVDs) represent a paramount global health concern, imposing substantial burdens on healthcare systems and affecting countless patients worldwide [1]. The management of CVD is often complex, particularly concerning medication regimens for patients with multiple comorbidities. Optimizing drug therapy in these scenarios is a cornerstone of effective treatment, yet it presents a significant and persistent challenge for healthcare providers.

In response to these challenges, the medical community has begun to explore the potential of large language models (LLMs) such as ChatGPT [2,3]. Initial studies have shown promise for LLMs in the CVD domain, where they can assist in analyzing patient-reported symptoms, generating differential diagnoses, and providing management plans in simulated cases that align with current medical knowledge [4,5]. Furthermore, research suggests that AI-driven platforms can offer valuable guidance to patients seeking information on CVD prevention, highlighting their potential as a supportive tool in patient care and underscoring their promise to revolutionize medicine [6,7,8].

However, despite this potential, the integration of LLMs into real-world clinical practice is fraught with significant concerns regarding their accuracy and reliability. Studies have revealed that while impressive in certain areas, LLM performance can be inconsistent [9,10]. These risks are not merely theoretical; a recent study evaluating LLM-simplified discharge summaries found that frequent omissions and critical hallucinations led to significant safety issues, with fewer than 60% of summaries being rated as completely accurate by physicians [11]. Crucially, while early CVD-related findings are encouraging, they often rely on simplified, simulated scenarios [5].

A critical research gap, therefore, exists regarding the reliability of LLMs in the nuanced, high-stakes domain of medication consultation for CVD patients with complex comorbidities. This is a domain where the combinatorial complexity of drug–disease interactions and polypharmacy [12] creates a significant challenge for general-purpose models, and where inaccurate advice could lead to severe adverse outcomes. To address this gap, the primary objective of this study is to simulate the real-world scenario of cardiologists using LLMs, in order to evaluate their potential and risks as a clinical decision support (CDS) tool. Our evaluation focuses specifically on the models’ performance when managing patients with complex multi-morbidities, with a critical emphasis on identifying whether they recommend contraindicated drugs. To this end, the study will make two main contributions:

1. Assess the consistency of AI-generated recommendations, both within a single model version and between different versions.
2. Determine the clinical validity and safety of these recommendations by measuring physician approval rates and systematically identifying any potentially contraindicated or inappropriate suggestions.

Through this rigorous evaluation, we aim to better delineate the boundaries for the safe and effective clinical application of this technology.

## 2. Method

### 2.1. Study Design

**Figure 1:**
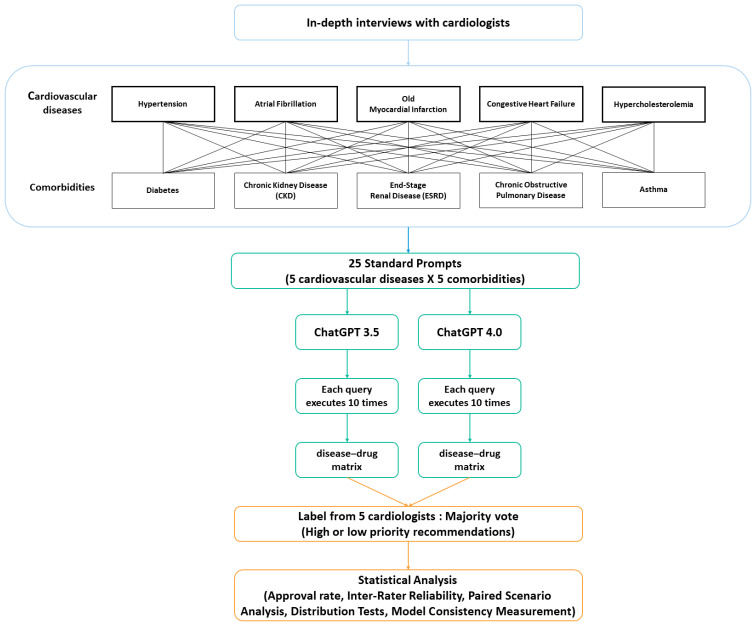
Flowchart of the study design.

In this project, we followed a multistep methodology for the sequential evaluation process shown in Figure 1. We began with in-depth interviews with cardiologists to identify the most common cardiovascular diseases in real clinical scenarios. Each physician provided detailed information on the incidence, common symptoms, and treatment methods based on their clinical experience. This information helped us determine the cardiovascular disease scenarios to be simulated in our study.

Based on the interviews, we identified the five most common cardiovascular diseases: hypertension, atrial fibrillation, old myocardial infarction, congestive heart failure, and hypercholesterolemia. Recognizing that patients with CVD often present with multiple comorbidities, we also selected five major conditions to create realistic clinical scenarios: diabetes, chronic kidney disease (CKD), end-stage renal disease (ESRD), chronic obstructive pulmonary disease (COPD), and asthma. These conditions were operationally defined based on established clinical guidelines, as detailed in Supplementary Table S1. These diseases and defined comorbidities reflect the clinical scenarios that cardiologists frequently encounter and serve as the basis for our evaluation of ChatGPT’s medication recommendations.

Following the identification of diseases and comorbidities, we constructed 25 distinct clinical scenarios by pairing each of the five CVDs with each of the five comorbidities. A standardized prompt structure was designed for all scenarios, exemplified by the following query: “If a patient has hypertension and diabetes (with no other comorbidities), what top 10 medications (with ATC code) would you recommend for treating hypertension?” A key methodological challenge was to balance the need for a controlled, reproducible experiment with the simulation of authentic clinical inquiries, which are often brief and do not include extensive patient data. Therefore, this simplified and standardized prompt structure was deliberately chosen to reflect such realistic usage patterns.

To assess the consistency and reliability of the responses, each of the 25 prompts was submitted 10 times to both ChatGPT models. All queries were executed between 21 January and 1 February 2024, using the platform’s default settings, which include a temperature of 1.0 and do not permit manual parameter adjustments by the end-user. To address the dual challenges of inherent model stochasticity and simulating a ‘naive’ clinical user who would not perform advanced prompt-engineering, this methodology was intentionally chosen. By maintaining a simple, direct prompt structure, our study evaluates the out-of-the-box performance of LLMs in these common scenarios. Consequently, the inherent response variability introduced by the default temperature setting is not treated as an experimental limitation but rather as a key component of our analysis, reflecting the unpredictability that clinicians would face in real-world applications.

### 2.2. Data Analysis

The dataset for this study was generated from 25 simulated CVD–comorbidity clinical scenarios. By querying both ChatGPT 3.5 and ChatGPT 4.0 ten times for each scenario, a total of 500 LLM-suggested medication responses were collected for analysis.

To establish a clinical ground truth for the AI-generated suggestions, a panel of five board-certified cardiologists was recruited to independently classify each unique medication recommendation. During this process, each physician assigned a drug to a category based on their clinical expertise and current treatment guidelines. The “High Priority” category was defined as first-choice treatments offering high efficacy and low risk. Conversely, the “Low Priority” category which included drugs less suitable due to side effects, lower efficacy, or lack of first-line recommendations was further stratified for a more granular analysis into three subcategories: (1) Low Priority: Maybe Useful, for drugs not considered first-choice but potentially effective in specific situations; (2) Low Priority: Not Useful, for drugs indicated for other conditions and unsuitable for the current scenario; and (3) Contraindicated, for drugs that must be avoided due to severe adverse effects or life-threatening complications. After each physician completed their independent classification, a final “ground truth” label for each drug was determined through a majority-vote process.

### 2.3. Performance Metrics

Several metrics were defined to quantitatively evaluate model performance. Model stability was assessed using two primary metrics: (1) response variability, quantified as the number of unique drugs recommended per scenario, and (2) intra-model response consistency, measured by the Jaccard similarity index. Therapeutic effectiveness was evaluated by two corresponding metrics: (1) the overall expert approval rate and (2) the proportion of high-priority recommendations.

### 2.4. Statistical Analysis

Inter-rater reliability among the five cardiologists was assessed using Fleiss’ Kappa to validate the expert-generated ground truth. To compare the primary outcomes between the two models, a two-proportion Z-test was used for the overall approval rates, and a chi-square (χ^2^) test was applied to the distribution of recommendations within the low-priority subcategories [13]. The non-parametric Wilcoxon Signed-Rank Test was employed for paired comparisons across the 25 scenarios to detect significant differences in response variability, intra-model response consistency, and the proportion of high-priority recommendations. For all inferential tests, a *p*-value of less than 0.05 was considered statistically significant.

## 3. Results

### 3.1. Baseline Model Characteristics and Rater Reliability

First, we characterized the baseline response patterns of the models. Across the 25 clinical scenarios, ChatGPT 4.0 exhibited slightly higher response variability (Mean = 14.84 unique drugs) compared to ChatGPT 3.5 (Mean = 12.44 unique drugs). Specifically, the number of unique drugs recommended by ChatGPT 3.5 ranged from 10 to 21, while the range for ChatGPT 4.0 was 10 to 25 (Table 1). However, a Wilcoxon Signed-Rank Test on the paired scores indicated this difference was not statistically significant (*p* = 0.220), suggesting a similar level of recommendation diversity between the models (Table 1). The complete list of medication recommendations generated for each scenario is available in Supplementary Table S2.

The reliability of the expert evaluation framework was then validated. The analysis of inter-rater reliability among the five cardiologists yielded a Fleiss’ Kappa of 0.536 (*p* < 0.001), indicating a moderate and statistically significant level of agreement. This confirms a reliable basis for the subsequent evaluation of the models’ performance.

### 3.2. Model Performance and Scenario-Specific Analysis

Our primary analysis focused on the clinical effectiveness of the models, beginning with an overall performance comparison. Based on the aggregated data from all 250 queries per model, ChatGPT 4.0 demonstrated a higher overall approval rate (86.90%) compared to ChatGPT 3.5 (85.06%). A two-proportion Z-test confirmed that this modest advantage was statistically significant (*p* = 0.0476), suggesting a general superiority of the newer model in providing clinically acceptable recommendations (Table 2).

**Figure 2:**
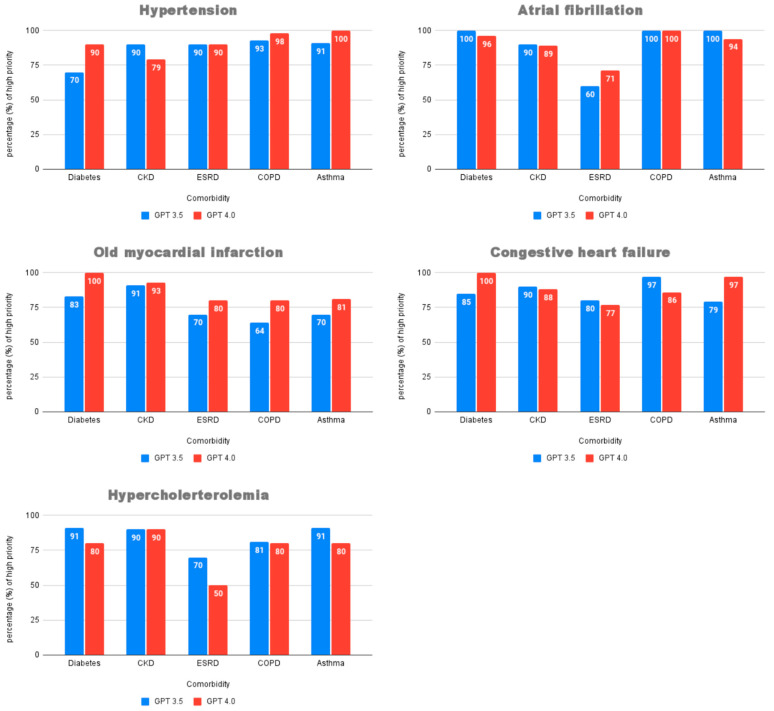
Proportion of high-priority medication recommendations by ChatGPT 3.5 and ChatGPT 4.0 in each disease–comorbidity scenario. Abbreviations: CKD, chronic kidney disease; ESRD, end-stage renal disease; COPD, chronic obstructive pulmonary disease.

However, to investigate whether this overall advantage was consistently maintained across the diverse clinical contexts, we performed a more rigorous, paired analysis on the proportion of high-priority recommendations for each of the 25 scenarios. The Wilcoxon Signed-Rank Test revealed no statistically significant difference between the two models in generating high-priority advice (Z = −0.830, *p* = 0.407). This crucial finding indicates that while GPT-4 may hold a slight edge when all data are pooled, its superiority is not systematic across individual clinical pairings. A detailed breakdown of performance in each scenario, as depicted in Figure 2, shows that while GPT-4 generally performed better in scenarios involving old myocardial infarction, its performance varied significantly in cases of hypertension, atrial fibrillation, and congestive heart failure.

Furthermore, an analysis of the less desirable recommendations revealed significant differences in the models’ error patterns. A chi-square test confirmed a significant difference in the distribution of recommendations across the three subcategories (“maybe useful”, “not useful”, and “contraindicated”) between the two versions (*p* < 0.001) (Table 3). This disparity was driven by ChatGPT 4.0 generating a higher frequency of “contraindicated” drugs, while ChatGPT 3.5 recommended more drugs that were “not useful” for the given scenario (i.e., indicated for other conditions) (Supplementary Figure S1).

**Figure 3:**
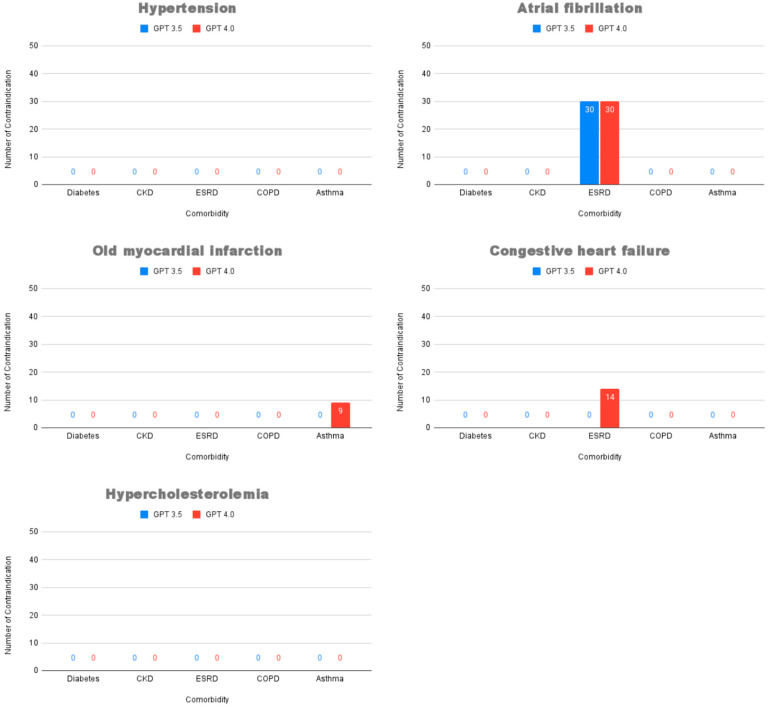
Number of medication recommendations classified as “contraindicated” between ChatGPT 3.5 and ChatGPT 4.0 across 25 cardiovascular disease–comorbidity scenarios. Abbreviations: CKD, chronic kidney disease; ESRD, end-stage renal disease; COPD, chronic obstructive pulmonary disease.

Of particular clinical concern is the generation of contraindicated drugs. As shown in Figure 3, both models recommended contraindicated medications in the high-risk scenario of atrial fibrillation with ESRD. Notably, ChatGPT 4.0, despite its higher overall approval rate, recommended contraindicated drugs with greater frequency and in more scenarios, including cases of old myocardial infarction with asthma and congestive heart failure with ESRD.

### 3.3. Model Consistency Analysis

**Figure 4:**
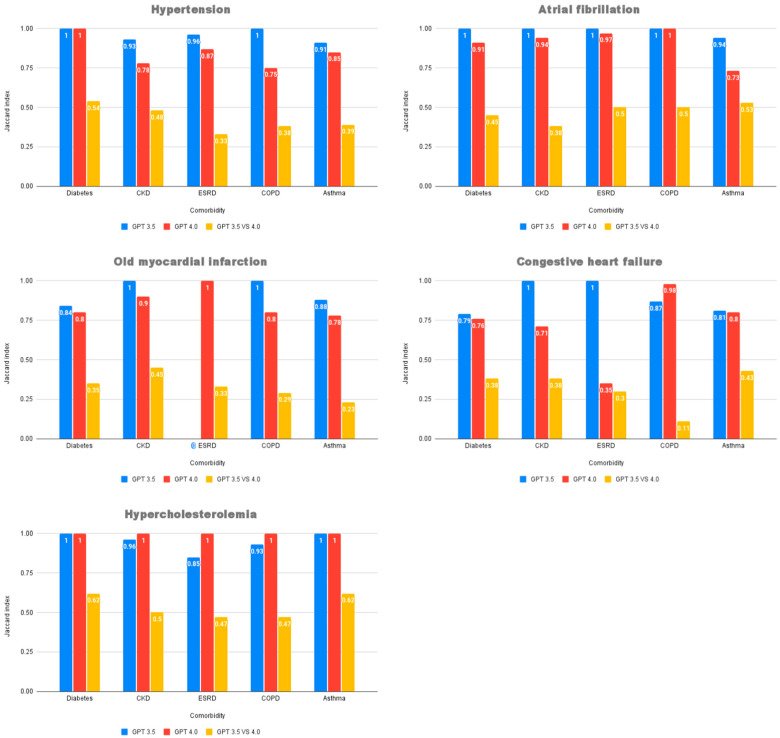
Jaccard Index Consistency for cardiovascular disease with comorbidity medication recommendations by ChatGPT 3.5 and ChatGPT 4.0 and the consistency between the recommendations of the two versions in each disease—comorbidity scenario. Abbreviations: CKD, chronic kidney disease; ESRD, end-stage renal disease; COPD, chronic obstructive pulmonary disease.

The intra-model consistency of recommendations was evaluated using the Jaccard similarity index (Figure 4). While ChatGPT 3.5 appeared descriptively more consistent, a Wilcoxon Signed-Rank Test showed no statistically significant difference in consistency between the two models (Z = −1.895, *p* = 0.058). Furthermore, the inter-model similarity was generally low, with a mean Jaccard index of only 0.42 (SD = 0.12) across all scenarios. This indicates that for any given condition, the two models often provided substantially different sets of recommendations.

## 4. Discussion

### 4.1. Principal Findings

This study is among the first to systematically evaluate ChatGPT’s performance in medication consultation for cardiovascular diseases (CVDs) complicated by comorbidities. Our findings reveal a nuanced picture: while the newer model, GPT-4, demonstrated a modest but statistically significant advantage in overall physician approval rates compared to GPT-3.5, this superiority was not consistently maintained when analyzed across specific, paired clinical scenarios. Most critically, our analysis identified significant safety concerns, as both models, particularly GPT-4, were prone to recommending contraindicated medications in high-risk clinical situations. This highlights a crucial gap between the general capabilities and the clinical-grade reliability of current large language models (LLMs).

### 4.2. Comparison with the Prior Literature

Our finding that model performance declines with increasing clinical complexity aligns with an emerging body of evidence. Prior research has shown high physician approval rates (e.g., 98.87%) for LLM recommendations in less complex, single-disease contexts [14]. In sharp contrast, our study’s lower overall rates (85–87%) in multi-morbid CVD scenarios underscore that clinical complexity is a primary challenge for LLM reliability. This decline is not merely a statistical artifact but reflects a fundamental limitation of general-purpose LLMs. As noted in recent reviews, these models are not yet mature enough to handle the combinatorial complexity of managing multiple chronic conditions. They struggle with the nuanced demands of polypharmacy and often provide overly simplified recommendations when faced with multi-morbidity [12,15]. Notably, despite the lower overall approval rate, ChatGPT demonstrated remarkable performance in certain instances. For example, in the scenario of atrial fibrillation with COPD, both models achieved a 100% approval rate for high-priority recommendations, suggesting that even in complex cases, perfect alignment with clinical consensus is possible. This exceptional performance, when contrasted with the significant variability observed across other scenarios, underscores that the model’s reliability is highly context-dependent.

### 4.3. Interpretation of Findings

The generation of contraindicated advice is the most significant clinical safety issue identified. Our study revealed specific instances of harmful recommendations, such as suggesting Dabigatran for patients with atrial fibrillation and end-stage renal disease (ESRD), despite its well-documented contraindication in this population [16,17]. Additionally, ChatGPT inappropriately recommended Carvedilol, a non-selective beta-blocker, for a patient with a history of myocardial infarction and asthma, a practice advised against by major clinical guidelines due to the risk of bronchospasm [18]. This erroneous recommendation likely stems from the limited clinical trial data and database information available for patients with comorbidity. This finding echoes concerns from other specialized fields, where LLMs have also shown low accuracy in complex cases like glomerular disease [19], despite performing well in other areas such as hepatocellular carcinoma management [20,21]. This suggests the risk is not isolated to cardiology but is a systemic issue in complex medical domains [22].

Furthermore, the response inconsistency we observed, both within each model and between the two versions, is consistent with findings from other researchers [23]. This variability likely stems from the inherent nature of LLMs themselves. As probabilistic “black box” models, their outputs are influenced by factors such as the randomness introduced by high temperature settings (a default for public-facing models), the specific composition and limitations of their training data (which may lack detailed information on multi-morbidity), and a lack of real-time knowledge updates to reflect the latest clinical guidelines [24,25,26,27]. These inherent limitations, particularly the ‘temporal misalignment’ of static training data with rapidly evolving clinical guidelines [28], point towards the need for new architectures. Retrieval-Augmented Generation (RAG) systems, which ground LLM outputs by retrieving information from trusted, up-to-date knowledge bases in real-time, represent a promising path forward to enhance both accuracy and consistency [29].

### 4.4. Strengths and Limitations

The primary strength of this study lies in its novel and clinically relevant design. By being among the first to systematically evaluate LLM performance in complex, multi-morbid CVD scenarios using a rigorous expert validation framework, we provide a crucial benchmark in a high-stakes medical domain. The reliability of this framework is evidenced by our inter-rater reliability analysis, which yielded a Fleiss’ Kappa of 0.536. This moderate level of agreement, rather than being a limitation, reflects the inherent nuances and valid differences in opinion that exist in complex clinical decision-making, a finding consistent with other similar studies [30,31]. This establishes that our evaluation is benchmarked against a realistic clinical consensus, not an artificial, perfect standard. Furthermore, our decision to focus on comparing two sequential, widely used versions of ChatGPT was deliberate. This approach was designed to reflect the actual usage patterns of clinicians, who often rely on the most accessible and prominent models, thereby enhancing the study’s real-world applicability [8,28,32].

Despite these strengths, several limitations must be acknowledged:

1. Snapshot in Time: Our evaluation is a snapshot of models from early 2024. Given the rapid evolution of LLMs, the specific performance metrics reported may not be generalizable to newer versions [33]. However, we believe our findings on the fundamental challenges, such as response inconsistency and the risk of contraindicated advice, remain highly relevant as benchmarks against which future models can be measured.
2. Limited Model Scope: This study focused exclusively on two versions of ChatGPT. While this was a deliberate choice to reflect real-world usage, a direct comparison with other contemporary models (e.g., Google’s Gemini, Anthropic’s Claude) was beyond the scope of this work and is an important area for future research.
3. Simplified Prompt Design: While we established clear, guideline-based definitions for comorbidities, our use of simplified prompts that did not include granular clinical details (e.g., specific eGFR values) was a deliberate methodological choice to simulate quick clinical queries. However, we acknowledge this approach creates a methodological trade-off. While it allowed for an effective evaluation of the models’ ‘out-of-the-box’ performance in realistic scenarios, the lack of specific data is also a limitation, as it may have constrained the models’ ability to provide more tailored recommendations and could have contributed to some of the observed inaccuracies [34].
4. Geographical and Sample Constraints: The study was conducted in Taiwan with a panel of five cardiologists. Although the medical practices and pharmaceuticals used are largely aligned with international standards, regional variations could limit the global generalizability of our specific findings.

## 5. Conclusions

This study provides a critical evaluation of ChatGPT’s readiness for complex cardiovascular medication consultation. Our findings demonstrate a crucial duality: while current LLMs can achieve high physician approval rates (over 85%), their performance is inconsistent and undermined by significant safety risks, including the generation of contraindicated advice. This level of reliability, while promising, does not yet match the near-perfect accuracy observed in less complex medical tasks, confirming that these models are not yet suitable for autonomous clinical application. Therefore, future work is essential to bridge this gap. Key research directions should include (1) leveraging real-world data, such as electronic health records and large-scale prescription databases, to validate and refine model performance, and (2) developing domain-specific models, fine-tuned on curated clinical guidelines to enhance both safety and accuracy. Until such advanced, safety-first systems are realized, vigilant professional oversight remains indispensable for any clinical use of this technology.

## Figures and Tables

**Table 1:** Response variability: number of unique drugs recommended across 10 repeated queries.

	Hypertension		Atrial Fibrillation		Old Myocardial Infarction		Congestive Heart Failure		Hypercholesterolemia	
	GPT 3.5	GPT 4.0	GPT 3.5	GPT 4.0	GPT 3.5	GPT 4.0	GPT 3.5	GPT 4.0	GPT 3.5	GPT 4.0
Diabetes	10	10	10	22	21	14	21	14	11	10
CKD	12	25	10	12	11	18	10	19	11	10
ESRD	11	13	10	11	10	10	10	20	16	10
COPD	14	15	11	10	14	13	16	14	12	10
Asthma	12	13	11	18	13	14	13	17	11	10

Comparison of the 25 paired scores was performed using a Wilcoxon Signed-Rank Test. Z = −1.227, *p* = 0.220. Abbreviations: CKD, chronic kidney disease; ESRD, end stage renal disease; COPD, chronic obstructive pulmonary disease.

**Table 2:** Approval rates and statistical comparison of the ChatGPT versions.

Version of the Model	“Low Priority” (n)	“High Priority” (n)	Total (N)	Approval Rate (%)
ChatGPT 3.5	404	2301	2705	85.06
ChatGPT 4.0	380	2521	2901	86.90

95% CI for the difference = 0.02% to 3.66%; Z = −1.981; *p* = 0.0476.

**Table 3:** Distribution of drug recommendations by subcategory and version with chi-square analysis.

	ChatGPT 3.5 (n = 2705)		ChatGPT 4.0 (n = 2901)	
	N	%	N	%
Low Priority: Maybe Useful	218	53.96	225	59.21
Low Priority: Not Useful	156	38.61	102	26.84
Contraindicated	30	7.43	53	13.95

*χ*^2^(df) = 17.0677 (2), *p* < 0.001. The column N represents the number of cases in each subcategory, while % indicates its proportion relative to the total cases. The chi-square (*χ*^2^) statistic, degrees of freedom (df), and *p* value are reported for the overall comparison.

## Data Availability

The original data and results presented in the study are included in the article and its Supplementary Materials.

## Supplementary Materials

The following supporting information can be downloaded at: https://www.mdpi.com/article/10.3390/healthcare13131598/s1, Table S1: Operational Definitions of Comorbidities Used in Study Scenarios. Table S2: Medication Recommendations for Cardiovascular diseases with comorbidities generated by ChatGPT 3.5 and ChatGPT 4.0. Figure S1: Number of medication recommendations classified as “low priority-not useful” between ChatGPT 3.5 and ChatGPT 4.0 across 25 cardiovascular disease-comorbidity scenarios.
